# Gut-brain axis in post-traumatic stress disorder: microbial - mediated mechanisms and new therapeutic approaches - A narrative review

**DOI:** 10.3389/fphar.2025.1621678

**Published:** 2025-06-25

**Authors:** Jiayue Pan, Shuairong Lin, Qiuling Qian, Shanni Fu, Xiaoliu Liu

**Affiliations:** ^1^ Brain Science and Advanced Technology Institute, Hubei Province Key Laboratory of Occupational Hazard Identification and Control, School of Medicine, Wuhan University of Science and Technology, Wuhan, Hubei, China; ^2^ School of Medicine, Wuhan University of Science and Technology, Wuhan, Hubei, China; ^3^ School of Life Sciences, Westlake University, Hangzhou, China

**Keywords:** posttraumatic stress disorder, gut microbiome, gut-microbiota-brain axis, vagus nerve, HPA axis, natural medicines

## Abstract

Post-traumatic stress disorder (PTSD) is a severe mental disorder that occurs after experiencing or witnessing a traumatic event. Not only does this disorder severely impair the quality of life and emotional wellbeing of patients, but in recent years the global rate of PTSD diagnoses has increased to 1.5–2 times, and the prevalence of PTSD associated with COVID-19 events in particular has surged to 10%–25%, underscoring the urgency of developing effective treatments. The lifetime prevalence of PTSD in the general population is estimated to be approximately 3.9%, while in high-risk populations, such as war veterans, it can be as high as 30%. As a key pathway connecting the central nervous system to peripheral organs, the gut-brain axis has received increasing attention for its role in PTSD. Although the gut-brain axis has been shown to be associated with several psychiatric disorders, especially depression, its specific role in PTSD remains undercharacterized. Existing studies suggest that specific strains of *Lactobacillus* (e.g., *Lactobacillus reuteri*) may alleviate inflammatory responses and improve PTSD-like behaviors by down-regulating the expression of pro-inflammatory factors (IL-6 and TNF-α). In this study, we used a narrative review approach to sort out the research progress of gut microbiota alteration in PTSD, and compared the characteristics of changes in specific microbial taxa (e.g., *Bacteroides*, *Lactobacillus*, *etc.*), the index of microbiota diversity (α/β diversity), and the levels of inflammatory markers (e.g., IL-6, TNF-α) between the animal model and the human patients, respectively, in order to We further explored the potential pathogenic mechanisms mediated by microorganisms, such as influencing the vagal pathway, hypothalamic-pituitary-adrenal (HPA) axis function, immune system and other processes involved in the pathology of PTSD, and summarized the intervention strategies targeting gut microecology, such as probiotic supplementation, dietary interventions and fecal bacteria transplantation.

## 1 Introduction

Post-traumatic stress disorder (PTSD) is a severe mental disorder characterized by re-experiencing of trauma, avoidance of trauma reminders, and hyperarousal symptoms that result in adverse emotional, cognitive, and physiological health responses (Compean and Hamner, 2019). Individuals with PTSD not only experience personal dysfunction (e.g., decreased occupational performance and broken family relationships), but also exhibit symptoms associated with depression, anxiety disorders, substance use disorders, and suicidal high levels of co-morbidity with ideation, resulting in a socioeconomic burden that far exceeds the direct medical burden (Davis et al., 2022; Kalisch et al., 2024). Currently, the conventional treatment of post-traumatic stress disorder (PTSD) consists mainly of pharmacologic and psychological interventions. In terms of medications, selective 5-hydroxytryptamine reuptake inhibitors (SSRIs) and norepinephrine reuptake inhibitors (SNRIs) are the first-line medications, but their efficacy is often limited, and some patients do not respond well to the medications, with problems such as a high relapse rate and pronounced side effects (Krediet et al., 2020). Psychological treatments such as cognitive behavioral therapy (CBT), although widely used, are also difficult to cover all patients due to treatment resources, compliance, and individual differences. Therefore, it is particularly urgent to explore alternative or complementary treatment strategies. In recent years, the gut-brain axis, as an important pathway regulating neuropsychiatric states, has gradually gained attention, providing new ideas for understanding the pathogenesis of PTSD and developing novel interventions.

Since Leclercq et al. suggested in 2016 that an early imbalanced gut microbiota may have long-lasting immune and other physiological effects that make individuals more susceptible to PTSD after traumatic events, attention has been focused on the role of the microbiota in PTSD and attempts have been made to manipulate certain gut bacterial communities to target PTSD (Chang et al., 2022; Leclercq et al., 2016; Petakh et al., 2024).

The gut microbiota, as a symbiotic system of trillions of microorganisms in the digestive tract, critically regulates central nervous system (CNS) function through the microbial-gut-brain axis (Cryan et al., 2019), and gut strains synthesize biologically active molecules such as neurotransmitters that directly intervene in brain activity and behavioral patterns, and whose metabolites enable individual and synergistic modulation of the immune response (Bremner, 2006; Dicks, 2022).

In this review, we first analyze the heterogeneity of gut microbiota and their common associations between animal models of PTSD and clinical patients, respectively, to reveal the ecological dysregulation patterns specific to this disease. Subsequently, the latest evidence on the influence of gut microbiota on the progression of PTSD is highlighted in terms of the classical pathological pathways of PTSD (e.g., dysregulation of the HPA axis, dysregulation of neurotransmitter signaling, dysregulation of the immune system, *etc.*). Finally, based on the theory of gut microbiota-PTSD interactions, innovative therapeutic strategies targeting microbiota modulation (e.g., probiotic interventions, fecal bacterial transplantation, and natural medicines targeting the gut-brain axis) are proposed.

## 2 Changes in the composition of gut microbiota in PTSD

### 2.1 Gut microbiota dysbiosis in people with PTSD

There was a significant difference in gut microbial diversity between people with PTSD and without PTSD (Table 1) (Núñez-Ríos et al., 2024).

**TABLE 1:** Summary of studies investigating gut microbiota in individuals with PTSD.

Study	Type of study	Population				Sample types	Sequencing platform	NOS score	Main findings	Limitations
		Sample size	Whether you are of white ethnicity	Gender, average age	Are you a high-risk occupational group?					
Bajaj et al. (2019)	Cohort study	93	No	Male veterans, mean age 58.8 ± 8.2 years	Yes	Fecal samples	16S rRNA gene sequencing	8	people with PTSD showed a significant decrease in microbiota diversity (Shannon index: 2.1 ± 0.5 vs. 2.5 ± 0.5 in the non-PTSD group, p = 0.03), and multiple regression confirmed that PTSD was an independent predictor (β = −0.28, p = 0.02). In terms of microbiota composition, the abundance of pathogens in the PTSD group increased: *Enterococcus* and *Escherichia/Shigella* increased	Sample was single sex (male), uncontrolled for diet, drug interference
Feldman R. et al. (2022)	Cohort study	232	No	All are mothers and children, age not specifically reported	No	Fecal samples	16S rRNA gene sequencing	9	The abundance of *Dialister* in the microbiota of people with PTSD was significantly reduced, while the abundance of *Veillonella* was increased (the key genus screened by the XGBOOST model, PTSD classification AUC = 0.84); the microbial synchrony between PTSD adolescents and their mothers was significantly reduced (the Euclidean distance of the microbiota composition increased, t = 1.97, p = 0.03); childhood PTSD symptoms directly predicted a decrease in the alpha diversity of the adolescent microbiota (Spearman correlation coefficient SCC = −0.49, p = 0.003)	Complex study design and weak functional analysis
Gao et al. (2022)	Cohort study	175	No	Age ranged from 18 to 50 years old, with no significant difference in gender	Yes	Fecal samples	16S rRNA gene sequencing	8	Long-term stress leads to persistent imbalance of microbiota. The α diversity of microbiota of frontline medical staff (FHWs) on the day when the anti-epidemic ended (Day 0) was significantly lower than that of second-line medical staff (SHWs) (Chao1 index: FHWs vs SHWs, P < 0.05), and the imbalance lasted for at least 180 days (the Simpson/Shannon index dropped to the lowest on Day 180, P < 0.0001). The abundance of anti-inflammatory bacteria such as *Faecalibacterium* and [*Eubacterium*] eligens group continued to decrease, which was positively correlated with depression and PTSD symptoms (random forest model importance score>0.3)	Lack of metabolome and animal experiments to validate the mechanism
Geier et al. (2024)	Case-control study	48	No	There was no significant difference in gender, with the PTSD group being 36.3 ± 16.1 years old and the control group being 44.9 ± 13.2 years old	No	Fecal samples	16S rRNA gene sequencing	7	The relative abundance of *Actinobacteria* was significantly reduced in the PTSD group (β = −0.50, *p* = 0.02), with an average log abundance of 8.5 in the control group vs 8.0 in the PTSD group The relative abundance of *Verrucomicrobia* was significantly increased in the PTSD group (β = +1.20, *p* <0.0001)	Small sample size, not included in analysis of gender differences
Levert-Levitt (2022)	cross-sectional study	189	No	Male, age not specified	Yes	Oral Saliva Sample	16S rRNA gene sequencing	-	PTSD severity (especially intrusive symptoms, excitatory/reactive symptoms) was significantly associated with a decrease in specific oral bacteria, mainly related to the following genera: *sp_HMT_914*, *sp_HMT_332*, *sp_HMT_871* and *Noxia* (FDR < 0.05). Differential abundance analysis (ANCOMBC) showed that the relative abundance of these genera was significantly reduced in people with PTSD and was directly associated with intrusive symptoms (such as flashbacks) and excitatory symptoms (such as irritability and sleep disorders). Increased abundance of *Bacteroidetes* was positively correlated with PTSD symptoms, and significantly increased in PTSD total symptom severity, reactive symptoms (such as hypervigilance), anxiety and memory impairment (FDR <0.05)	Non-invasive sampling may confound dietary, oral hygiene factors
Zhou et al. (2021)	Cohort study	29	No	The majority of the patients were young women, with the rehabilitation group aged 29 and the control group aged 37.5	Yes	Fecal samples	16S rRNA gene sequencing	6	The microbiota of recovered COVID-19 medical staff (3 months after discharge) was significantly different from that of the healthy control group, which was manifested by reduced microbiota diversity (Shannon index decreased significantly, p = 0.016), and changes in the abundance of specific bacterial genera were significantly correlated with persistent symptoms: (1) Enrichment of opportunistic pathogens: ① Unclassified *Escherichia coli* was positively correlated with fatigue (r = 0.567, p = 0.028), chest tightness after activity (r = 0.687, p = 0.005), and myalgia (r = 0.523, p = 0.045). ② *Pasteurella* enterica was positively correlated with anorexia (r = 0.629, p = 0.012) and fatigue (r = 0.545, p = 0.036) (2) Decrease in beneficial bacteria: ① *Faecalibacterium prausnitzii* was negatively correlated with chest tightness after activity (r = −0.591, p = 0.02). ② Butyric acid-producing Enterobacteriaceae was negatively correlated with cough (r = −0.635, p = 0.011)	Very small sample size, longer-term colony recovery not tracked
Malan-Muller et al. (2022)	Case-control study	137	No	Community sample of predominantly mixed-race South African women, average age 44	No	Fecal samples	16S rRNA gene sequencing	7	The random forest model identified a microbial consortium consisting of four genera, *Mitsuokella, Odoribacter, Catenibacterium,* and *Olsenella*, that could distinguish PTSD from controls with 66.4% accuracy (33.6% error rate). This consortium was significantly more abundant in the PTSD group (p < 0.05) and was positively correlated with PTSD severity (CAPS-5 total score) (Spearman r = 0.25, p = 0.003) and childhood trauma (CTQ total score) (r = 0.17, p = 0.05)	Effects of uncontrolled psychotropic drug use on bacterial microbiota
Hemmings et al. (2017)	Case-control study	30	No	30 South African mixed-race adults (77% female), average age about 40 years, people with PTSD were mainly female (78%)	No	Fecal samples	16S rRNA gene sequencing	5	We found that in a South African mixed-ancestry population, there was no significant difference in the overall diversity of the gut microbiota (α/β diversity) between people with PTSD and trauma-exposed controls (TE), but a significant decrease in the relative abundance of specific bacterial phyla Actinobacteria, Lentisphaerae, and Verrucomicrobia was associated with PTSD severity: the relative abundance of *Actinobacteria*, *Lentisphaerae*, and *Verrucomicrobia* was negatively correlated with PTSD symptom severity (CAPS-5 total score) (Pearson’s r = −0.387, P = 0.035)	The sample size was very small and the results were not replicated in other populations
Malan-Müller et al. (2023)	Case-control study	198	Yes	Most of the respondents are Spanish young women (70%), aged between 30 and 43 years old	Yes	Fecal samples	16S rRNA gene sequencing	7	The relative abundance of *Turicobacter* *sanguinis* was significantly increased (after GLM correction: p = 0.0008, effect size r = 0.24) The relative abundance of the phylum Lentisphaerae was significantly reduced (after GLM correction: p = 0.002, effect size r = 0.20)	Cross-sectional design, lack of causal validation
Commonality				Targeting trauma-exposed populations (veterans, healthcare workers, *etc.*)		Fecal samples predominate, but oral samples are present	16S rRNA gene sequencing		microbiota heterogeneity may mediate symptoms through the gut-brain axis, with specific genera repeatedly validated as biomarkers (e.g., *Enterococcus,* Trichosporonaceae), and the majority of studies have found that PTSD is associated with reduced microbiota diversity and increased pro-inflammatory genera	Sample sizes are generally small, mechanism validation is inadequate, and most are cross-sectional studies
Unique characteristic				Association of different symptom typing with the bacterium genus (Literature 4, 7)		Oral samples are present	Partially bound animals Experiments (Literature 2)		Predictive value of colonization for PTSD and causality of colonization transplantation Validation as a unique contribution, with different studies focusing on different colonization sites (microbiota/oral) and populations (cirrhosis, COVID-19 survivors, general trauma)	Limitations vary (e.g., gender restrictions, metabolomics not included, experimental design differences)

The risk of bias was assessed by evaluating selectivity, comparability, and outcome. The selection of research subjects was scored four points according to (① whether the case was appropriately determined, ② the representativeness of the case, ③ the selection of the control, and ④ the determination of the control), and the comparability was scored two points according to (considering the comparability of cases and controls in the design and statistical analysis to control confounding factors), and the exposure factor measurement was scored three points according to (① determination of exposure factors, ② using the same method to determine the exposure factors of cases and controls, and ③ non-response rate), for a total of nine points. 0–4 points were low-quality studies, and 5-9 points were high-quality studies.

Abbreviations: PTSD: Post-Traumatic Stress Disorder; FHW: frontline health workers; SHW: secondary health workers; AUC: area under curve; NOS: Newcastle-Ottawa Scale.

Hemmings et al. used the DSM-5 PTSD scale to diagnose PTSD, extracted microbial DNA from stool samples of PTSD individuals and 12 TE control participants, generated bacterial 16S ribosomal RNA gene V3/V4 amplicons, and sequenced them, and although randomized forest analyses indicated that the total abundance (The total relative abundance of specific taxonomic units detected in a given sample, which equals to relative abundance in this paper) Actinobacteria, *Microbacterium verticillioides*, and Lentisphaerae was significantly negatively correlated with PTSD symptoms, alpha diversity and beta diversity of microorganisms of this genus were found to be significantly higher in people with PTSD and without PTSD (Hemmings et al., 2017). Although Random Forest analysis showed a significant negative correlation between the total abundance of Actinobacteria, *Micrococcus verticillioides*, and Lentisphaerae and PTSD symptoms, suggesting a detrimental effect of this genus of microorganisms on the PTSD population, the alpha and beta diversity of the microorganisms revealed that the overall microbial diversity (in the original language, the correct term should be “relative abundance”) measurements were similar between the PTSD and TE controls, a discrepancy which may be due to the very small sample size. A methodologically similar study was conducted by Malan et al. who also examined the association between gut microbiota composition and PTSD outcomes using a randomized forest model among participants, selecting and identifying four genera that were able to differentiate between the PTSD and TEC groups, i.e., Mitsuokella, Odoribacter, Catenoidea, and *C. elegans*, as well as the PTSD and TEC groups. Odoribacter, Catenibacterium, and Olsenella, and the relative abundance of these genera was higher in people with PTSD compared with controls and positively correlated with CAPS-5 scores (Malan-Muller et al., 2022), suggesting a pathogenic role of these four genera in PTSD. In addition, another Mendelian randomization study found a significant negative correlation between *Micrococcus luteus* and the risk of post-traumatic stress-related depression (PTMDD), suggesting a protective effect of *Micrococcus wartyi* against PTMDD (S. Liu et al., 2024). At the level of microbiota categorization, it has been shown that individuals with PTSD generally exhibit reduced levels of butyric acid-producing anti-inflammatory strains (e.g., *Enterococcus faecalis*) and significant enrichment of pro-inflammatory strains (e.g., *Eggerthella*). This bacterial imbalance may be closely related to the impaired anti-inflammatory function and activation of the inflammatory cascade response, which will be mentioned later, and constitutes an important pathological basis for the pathogenesis of PTSD (Nikolova et al., 2021). Future studies need to further clarify the potential mechanisms of damage by pro-inflammatory microbiota on the pathologic state of PTSD in order to reveal its complex role in disease development.

In addition to sample size, selection of study population, source of biological samples are important factors in determining the results of microbiota studies. Bajaj et al. (2019); Feldman E. L. et al. (2022) explored the association between PTSD and gut microbiota, with the former using adult male veterans with cirrhosis of the liver, and found that people with PTSD had decreased gut diversity and accompanied by an increase in pathogenic bacteria. In contrast, Feldman et al. used a broader population (including mothers and infants) to validate the causal relationship between gut microbiota and anxiety behaviors through a colony transplantation experiment, which strengthens the biological explanatory power of the study. For biological samples, most studies used fecal samples (e.g., Hemmings, Geier, Malan-Muller) (Geier et al., 2024; Hemmings et al., 2017; Malan-Muller et al., 2022), which are convenient to reflect the overall structure of the gut microbiota. Levert-Levitt et al. (2022) used oral saliva samples, and found that specific oral bacterial genera were correlated with the severity of PTSD, but because oral microbiota is more susceptible to diet, hygiene, and smoking, their conclusion that oral microbiota characteristics (decrease in HMT_914 and increase in *Mycobacterium avium* phylum) correlate with PTSD severity remains to be tested. Exposure to chronic stress-induced PTSD is also influenced by the role of the gut-brain axis, and a study on the relationship between gut microbial ecology and secondary PTSD in frontline healthcare workers in the New Crown epidemic reported that chronic exposure to stressful and anxious frontline work environments resulted in significantly lower individual counts of each species of gut microbiota among frontline workers than among secondary healthcare workers, and demonstrated sustained 6-month follow up changes (Zhang et al., 2023). 16S rRNA gene sequencing longitudinal analysis revealed that high abundance of *Ehrlichia* anomalies was an important determinant of the recurrence of post-traumatic stress symptoms in frontline healthcare workers, and that a series of stressful events fighting COVID-19 induced characteristic longitudinal changes in the gut microbiota, which underlie the changes in the dynamic mental state (F. Gao et al., 2022). In addition, the gut microbiota of COVID-19 convalescent patients 3 months after discharge from the hospital differs from that of healthy individuals, with increases in unclassified *Escherichia coli*, *Enterobacter* pasteurianus, and Enteromonas acidophilus butyric acidophilus associated with persistent postdischarge symptoms (Zhou et al., 2021). These findings suggest that future PTSD and microbiota-related studies need to continue to advance in terms of more rigorous study design, more scientific sample selection, and more in-depth biological mechanisms, with a view to establishing more robust microecological intervention strategies to serve the treatment of precision psychiatric disorders.

### 2.2 Gut microbiota dysbiosis in PTSD animal models

Animal models are a valuable addition to human studies compared with populations, and most studies on the microbiota composition of animals have explored genus-level changes in dexposed mice infected with the gut pathogen *Citrobacter* rodentium to acute stress showed significant memory dysfunction (Gareau et al., 2011), and Yang et al. found in a model of chronic social defeat stress (CSDS) that stress-resistant mice had a significant enrichment of Bifidobacterium bifidum in their intestines (Yang W. T. et al., 2017). *Bifidobacterium* were significantly enriched in the intestine, as well as Actinobacteria, suggesting that the rise in gut microbiota may affect memory, behavior after acute and chronic stress.

Szyszkowicz et al. reported that the genus Turicibacter showed a decreasing trend in a model of chronic social frustration, whereas the genus Flavonifractor was significantly elevated (Szyszkowicz et al., 2017). These genera all belong to the phylum Firmicutes, suggesting that there is also structural remodeling within this phylum within the microbiota under stress. In the same vein, a novel animal model of PTSD based on arousal-based individual screening (AIS) demonstrated an increase in the genera Tuzzerella and Lachnospiraceae NK4A13 in the family Lachnospiraceae, but Tyzzerella, Lachnospiraceae UCG 010, and Lachnospiraceae FCS020 groups decreased (Laudani et al., 2023), also suggesting structural remodeling within the clade under stress. Meanwhile, studies using the classical animal model of PTSD with single-time prolonged stress (SPS) showed that the abundance of *Roseburia*, *Oscillibacter* and *Trichoderma* unspecified in the PTSD group was also significantly lower than that in the control group (Tanelian et al., 2023), all suggesting that stress affects the decline in the level of gut microbiota in the animals. Ipth, allowing us to assign animal controls to trauma-exposed groups and assess the gut microbiota before and after trauma (Ke et al., 2023). For example, studies that en addition, more and more studies have confirmed that the synthesis of key mediators of the gut-brain axis can be induced by the use of probiotics (*Bifidobacterium longum*, *Lactobacillus mucosus*) to regulate the occurrence of stress behaviors, whose mechanisms will be introduced in the subsequent content (Bercik et al., 2011a; Bercik et al., 2011b; Han and Kim, 2019) (Table 2).

**TABLE 2:** Preclinical (murine) studies investigating gut microbiota and PTSD.

Study	Animal models	Stressor	Sample sizes	Sequencing platform	Main findings		
					Main findings	Changes in behavioral indicators	Diversity at the phylum level of microbiota
Yang C. et al. (2017)	Male C57BL/6 mice	Chronic Social Defeat (CSDS)	8 in the susceptible group, 6 in the recovery group and 8 in the control group	16S rRNA (V4 region) T-RFLP technology	Significant enrichment of microbiota *bifidobacteria* in stress-resistant mice; oral supplementation enhances stress resistance	Oral supplementation with Bifidobacterium increased the social interaction time (in the presence of the attacking mouse) of CSDS mice from 40 to 80 s (p < 0.05), and the sucrose preference rate from 55% to 75%	This study used the CSDS model to find that the proportion of *bifidobacteria* in the microbiota of mice in the stress-resistant group was significantly increased (1.5%–2.0%, p < 0.01), while it was not detected in the control group and the susceptible group (p < 0.001), proving that *bifidobacteria* are key microorganisms that mediate stress resilience, and their intervention can prevent depressive-like phenotypes
Gautam et al. (2018)	Male C57BL/6J mice	Social defeat stress model	There were 7 rats in the control group and 8 rats in the stress group	16S rRNA gene V3-V4 hypervariable region	PTSD-like social stress can rapidly trigger microbiota imbalance within 24 h, which is characterized by a sharp imbalance in the ratio of Firmicutes to *Bacteroidetes* (F/B value) (Firmicutes increased significantly and *Bacteroidetes* decreased significantly), accompanied by a continuous decrease in the abundance of Verrucomicrobia and dynamic fluctuations in key functional bacterial genera (such as Akkermansia and *Lactobacillus*)	The weight of mice in the attacker-exposed group (Agg-E) was significantly higher than that in the control group after 10 days of stress. Correlation analysis showed that there was no statistical correlation between weight change and the ratio of Firmicutes/*Bacteroidetes*. Other indicators were not reported	The proportion of Firmicutes in the stress group increased to 69% (vs. 59% in the control group), and the proportion of *Bacteroidetes* decreased to 13% (vs. 25%), resulting in a surge in the F/B ratio from a baseline of 2.3–11.2 within 24 h of exposure (p < 0.001). The abundance of Verrucomicrobia was significantly reduced (logFC = −2.5 on day 4, p < 0.001)
Bharwani et al. (2017)	Male C57BL/6 mice	Chronic social frustration	Control group: received PBS gavage; treatment group: received lactic rod gavage	16S rRNA	*Lactobacillus rhamnosus* JB-1 improves anxiety and social deficits and modulates dendritic cell/Treg activity	The mice in the stress control group showed significant social avoidance (interaction effect of time in social chamber and non-social chamber: (F_1,23_ = 5.438, p = 0.029), while the JB-1 treated group (DEF/JB-1) showed no such avoidance (p > 0.05), and its social/non-social time ratio was significantly higher than that in the stress control group (F_1,39_ = 9.660, p = 0.004)	a significant decrease in the phylogenetic diversity (F_1,68_ = 13.21, p = 0.0005) and Chao1 richness (F_1,68_ = 12.50, p = 0.0007) of the mouse gut microbiota. This disruption persisted for at least 3 weeks after the stress ended (phylogenetic diversity: F_2,28_ = 7.893, p = 0.002; Chao1 richness: F_2,28_ = 6.061, p = 0.007)
Diaz Heijtz et al. (2011)	Male NMRI mice	Gut microbial deficiencies	SPF group (normal colony mice), GF group (germ-free mice), CON group (GF mice with early colony colonization)	16S rRNA sequencing	Hyperactivity/hypoanxiety in GF mice; early colony colonization reversal of synaptic gene (PSD-95) abnormalities	GF mice showed significantly enhanced locomotor activity and reduced anxiety-like behavior. In the open field test, the total movement distance of GF mice was significantly higher than that of SPF mice (p < 0.05), and the slow (>5 cm/s) and fast (>20 cm/s) movement time in the late test period (20–60 min) were significantly increased (p < 0.05). In the anxiety behavior test, GF mice stayed in the light area of the light-dark box for a longer time (P < 0.05)	-
Pearson-Leary et al. (2020)	Male SD rats	Social frustration	Social frustration modeling experiment: control group n = 8, short latency/vulnerable group n = 9 long latency/resistant group n = 10	16S rRNA sequencing	Stress-sensitive microbiota induces IL-1β ↑ and depressive-like behavior in receptor hippocampal region	Social interaction test: There was no statistical difference in interaction time between the recipient groups transplanted with SL/susceptible rat microbes (n = 11–14) and the recipient groups transplanted with LL/resistant rat microbes, the non-stressed control group, or the PBS vehicle group, indicating that the transplantation did not induce anxiety-like behavior	Microbial microbiota characteristics after stress: After 7 days of social defeat stress, the α diversity of microbiota in SL/vulnerable rats increased significantly (p = 0.003), while there was no such change in the control group and stress-resistant rats. The relative abundance of *Clostridia* in the SL group was significantly higher than that in the control group (p = 0.05), and the abundance of *Clostridium* increased (p = 0.02). The abundance of *Bacteroidetes* was significantly reduced (p = 0.004), and the ratio of Firmicutes to *Bacteroidetes* (F/B ratio) increased in both stress groups (p < 0.05). The abundance of Actinobacteria in the SL group was higher than that in the control group (p = 0.04) and the LL group (p = 0.05)
Szyszkowicz et al. (2017)	Male C57BL/6 mice	Chronic social frustration	Stress group 24	16S rRNA sequencing	Social Avoidance Severity Correlates with *Flavobacterium↑/Turicibacter* ↓ and Prefrontal IL-1β/IL-6 ↑	After 10 days of social defeat stress, 55.6% of the mice (10/18) showed a “susceptible phenotype”, with their social interaction ratio significantly reduced to 0.57 ± 0.09 (control group: 1.59 ± 0.06; p < 0.0001), accompanied by significant social avoidance behavior (such as reduced contact time with the target mouse and increased corner residence time)	Three weeks later (i.e., a lasting effect after the stress ended), the cecal microbiota of these susceptible mice showed specific changes
Tanelian et al. (2023)	Female SD rats	Single Prolonged Stress (SPS)	Control group 10 SPS Experimental group 16	Illumina platform for 16S V3-V4 rDNA sequencing	Disturbed microbiota in SPS-sensitive individuals is accompanied by ↑ blood-brain barrier (BBB) leakage and ↑ neuroinflammation, with a dynamic microbiota-phenotype association	Social deficits: The duration of active social interaction in the SPS-S group was only 41.5 ± 5.2 s (vs 82.3 ± 6.1 s in the SPS-R group, p < 0.01), and the number of times of initiating social interactions was 12.4 ± 1.8 times (vs 25.6 ± 2.3 times in the SPS-R group, p < 0.01), indicating significant social avoidance	After SPS exposure, the α diversity of the microbiota in the SPS-S group increased significantly (Chao1 index: SPS-S was 25% higher than the control group, p < 0.05), but was accompanied by the enrichment of harmful bacterial genera
Kelly et al. (2022)	Male SD rats	Trauma + chronic restraint stress	32 rats	16S rRNA gene V3–V4 region	Chronic restraint stress (rather than simple trauma) is the core factor driving microbiota disturbance. Continuous stress leads to abnormal increase in microbiota α diversity and significant changes in key bacterial genera, including explosive proliferation of *Bacteroidetes* and irreversible loss of Actinomycetes	No behavioral data were measured	*Bacteroidetes*: ①LCHS/CS 7/7 group: relative abundance increased from 0.01 at baseline (POD 0) to 0.04 at POD 7, and returned to baseline at POD 14; LCHS/CS 14 group: relative abundance increased from 0.005 at baseline to 0.015 at POD 7 Actinomycetes: LCHS/CS 7/7 group: the abundance completely disappeared after POD 7 and did not recover on POD 14 The abundance of *Clostridium*: LCHS/CS group 14: C. celatum and other species continued to decrease after POD 0

Abbreviations: CSDS: Chronic Social Defeat; DSS: dextran sulfate; T-RFLP: Terminal-restriction fragment length polymorphism; BDNF: brain-derived neurotrophic factor; PBS: phosphate buffered saline; SPF: specific pathogen free; GF: germ-free; BBB: Blood-brain barrier; LCHS: pulmonary contusion + hemorrhagic shock; LCHS/CS, 7/7 group: LCHS +7 days of restraint stress +7 days of conventional feeding; LCHS/CS, 14 group: LCHS +14 days of continuous restraint stress.

## 3 Interaction between PTSD pathogenesis and gut microbiota

**FIGURE 1:**
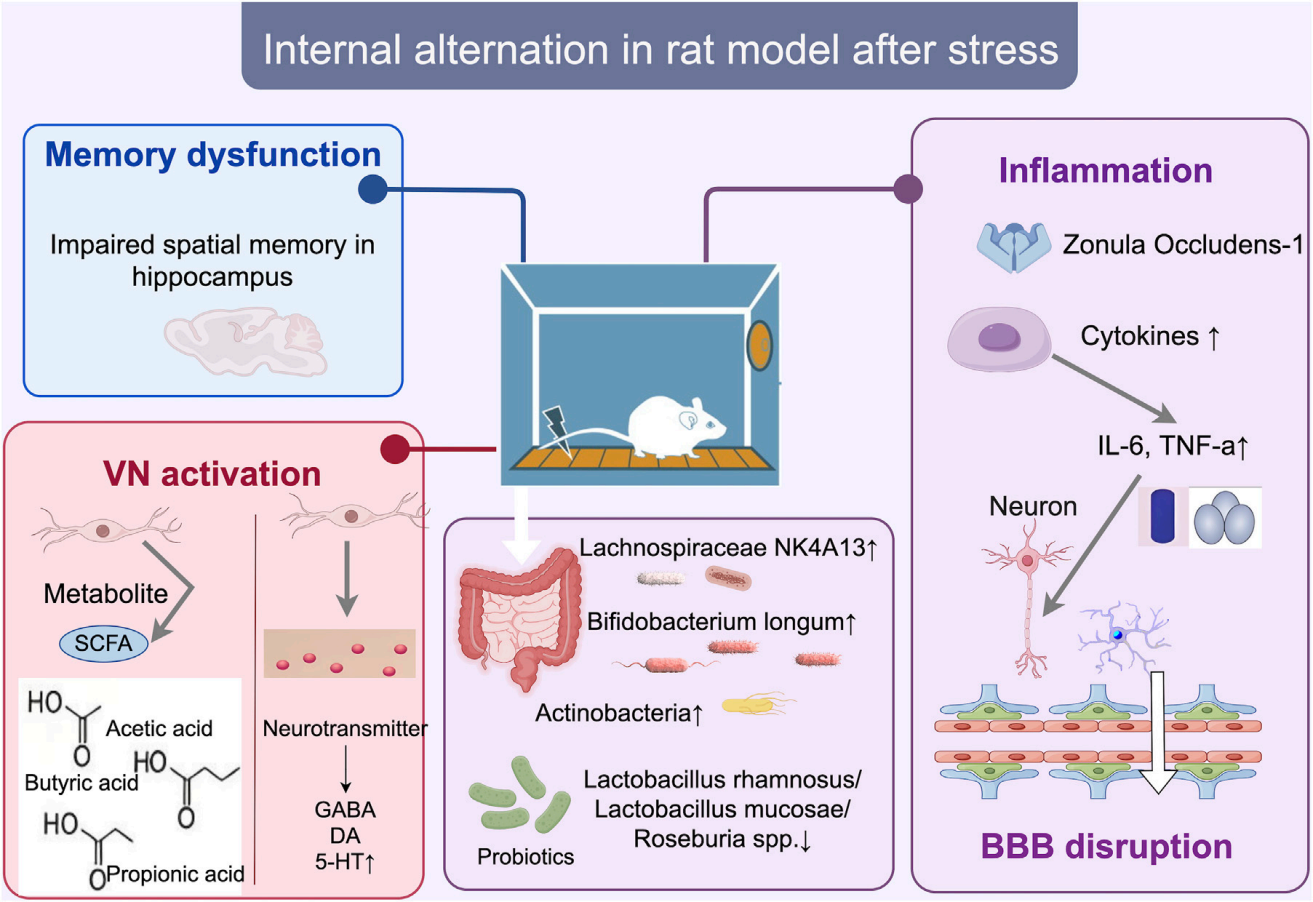
Internal changes in the SD rat model of stress disorder. After conditioned fear training, bacterial microbiota such as Lachnospiraceae NK4A131, Bifidobacterium longum, and Actinobacteria increased, triggering a systemic inflammatory response (increase in IL-6, TNF-a), which may directly affect brain regions such as the hippocampus, where memory deficits occur, and inducing ZO-1 proteins and cytokine Disrupts the blood-brain barrier (BBB) and neurons or microglia, and activates vagal pathways, increases colony metabolites SCFA (acetic acid, butyric acid, propionic acid), and induces neurotransmitter changes (increase in 5-HT and decrease in GABA). bbb, blood-brain barrier; zo-1, Zonula Occludens-1 (Zonula Occludens-1).

**FIGURE 2:**
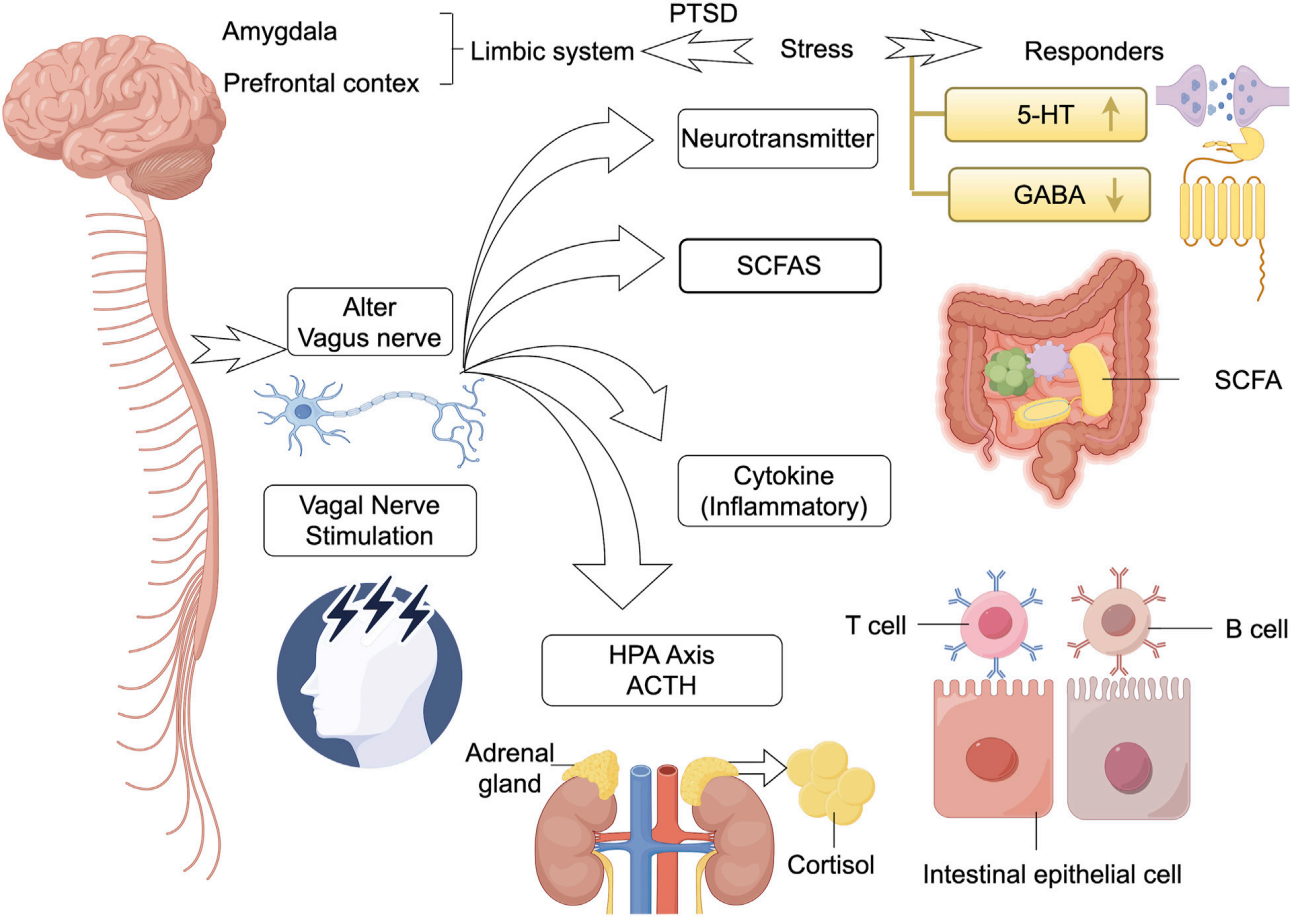
Interplay and connections inside brain-gut microbiot. The gut microbiota and its metabolites (e.g., SCFAs) communicate bi-directionally with key brain regions (e.g., prefrontal cortex, amygdala, limbic system) through multiple pathways (including the vagus nerve, the HPA axis, the immune system, and neurotransmitters). Stimulation of the microbiota can lead to hyperactivation of the HPA axis (involving ACTH, cortisol), dysbiosis levels of neurotransmitters (e.g., 5-HT, GABA), T/Bcell activation, and changes in the function of gut epithelial cells, which collectively contribute to the stress dysregulation and pathology of PTSD. In addition, vagus nerve stimulation has been shown to be effective in reversing such brain-gut reactions. HPA, hypothalamic-pituitary-adrenal; SCFA, chain fatty acids; 5-HT, 5-hydroxytryptamine; GABA, gamma-aminobutyric acid; ↑ represents the increased levels of this neurotransmitter.

The pathogenesis of PTSD involves a complex interplay of biological, psychological and environmental factors (Settanni et al., 2021). Its classical pathogenesis is mainly related to vagal dysfunction, neurotransmitter imbalance (Shin and Liberzon, 2010), hypothalamic-pituitary-adrenal axis (HPA axis) dysregulation and immune system dysfunction (Bandelow et al., 2017; Schumacher et al., 2019) (Figure 1). The gut microbiota affects the nervous system through the above regulatory mechanisms (Rieder et al., 2017). At the same time, gut microbiota can also regulate stress, providing new therapeutic targets for the treatment of PTSD (Leclercq et al., 2016) (Figure 2).

### 3.1 Vagal pathways

The gut microbiota is able to act on the vagus nerve and transmit this information to the CNS, which directly activates neurons in the brain (Eisenstein, 2016; Fülling et al., 2019), thereby altering host behavioral outcomes. It was found that when mice were infected with subclinical doses of *Campylobacter* jejuni, by activating neurons within the solitary tract nucleus of the vagus nerve, the mice began to exhibit more anxiety-like behaviors (Goehler et al., 2005). Conversely, ingestion of *Lactobacillus rhamnosus* can modulate anxiety *via* the vagus nerve thereby treating PTSD (Bravo et al., 2011).

The vagus nerve, as the main neural pathway connecting the brain and visceral organs, regulates the activity of the autonomic nervous system in people with PTSD and modulates the stress response (Q. Zhou et al., 2020). Specifically, the vagus nerve acts to restore homeostasis in the body after stress (Costantini et al., 2010). It reduces heart rate and blood pressure and lowers cortisol levels, thereby reducing the effects of trauma (Almeida et al., 2021). In patients with PTSD, both vagal tone and heart rate variability (HRV, an indicator of parasympathetic activity) are significantly reduced, and increased vagal activity is associated with a reduced risk of developing PTSD (L. J. Noble et al., 2019). Non-invasive percutaneous auricular vagus nerve stimulation is increasingly recognized as a promising approach for the treatment of PTSD. For example, electrical stimulation of vagal afferent fibers alters neurotransmitter levels in the brain (Schumacher et al., 2019). This improves heart rate variability and attenuates symptoms of persistent hypervigilance in people with PTSD, thereby significantly reducing their emotional and somatic symptoms (N. C. Noble et al., 2023).

In addition, Liu et al. showed that a lack of vagal integrity also interrupts the immune component of the microbiota-gut-brain axis, which inhibits the effects of *L*. *rhamnosus* on behavioral and cortisol stress responses (Y. Liu et al., 2021). Animal models of PTSD have shown that elevated vagal tone is also accompanied by dysregulation of the HPA axis, alterations in the neurotransmitter system, and immune dysregulation (Cohen and Zohar, 2004). During stress, the vagus nerve inhibits m1-type pro-inflammatory macrophages, whose anti-inflammatory effects alter gut permeability and gut microbiota (Bonaz et al., 2017). Impairment of vagal tone reduces the body’s ability to suppress the hyperinflammatory response that is a key factor in the chronic stress response in PTSD (Dabrowska, 2023). This inflammatory imbalance may further lead to neurobiological changes that reinforce adverse fear responses and emotional susceptibility after stress (Breit et al., 2018). In summary, the vagus nerve plays a central role in the bidirectional neuroimmunoendocrine pathway of the microbiota-gut-brain axis.

### 3.2 HPA axis dysregulation

Changes in the composition of the gut microbiota may lead to dysregulation of the HPA axis, which in turn affects the neuroendocrine system of the brain (Sudo et al., 2004). GF mice have significantly higher elevations of plasma adrenocorticotropic hormone and corticosterone in response to inhibitory stress than mice without specific pathogens, and this HPA stress response can, in turn, be reversed by reconstituting *bifidobacteria* at a young age (Huo et al., 2017). Prebiotic and probiotic interventions have emerged as potential strategies for PTSD treatment. Animal experiments have shown that *Lactobacillus farciminis* attenuates acute psychological stress in rats by modulating hypothalamic adrenocorticotropin-releasing hormone gene expression, decreasing adrenocorticotropic hormone and cortisol secretion, preventing gut barrier damage, and decreasing circulating LPS levels (Ait-Belgnaoui et al., 2012). The GF animal model has shown that the microbiota influences the stress and stress response in rats by modulating the baseline of HPA activity and by lowering adrenocorticotropic hormone and cortisol levels to influence stress and anxiety-like behaviors (Hendrickson and Raskind, 2016). Probiotics have been shown to be effective in the treatment of PTSD with cortisol levels (Pivac et al., 2023).

Animal and population studies have confirmed the presence of HPA axis dysfunction in patients with PTSD (Bremner, 2006), with elevated levels of adrenocorticotropin-releasing hormone and significantly lower basal cortisol levels, leading to abnormal stress responses (Dunlop and Wong, 2019; van der Kolk et al., 1985). Low cortisol levels not only make individuals more susceptible to PTSD after traumatic events (Leclercq et al., 2016), but also alter gut barrier function: increasing gut permeability (“leaky gut”), facilitating the entry of bacteria and their products into the mucosal layer, and triggering inflammation (Rudzki et al., 2017). Thus, gut microbiota not only regulates the stress response of the HPA axis, but is also inversely regulated by the HPA axis (Bailey and Coe, 1999), and glucocorticoids, mainly cortisol, are also immunosuppressive and may disrupt gut immune homeostasis (Van Wyngene et al., 2021). Dysregulation of the neuroendocrine system has been shown to be an important feature of patients with PTSD (Foster et al., 2017).

The HPA axis influences immune and inflammatory responses not only because cortisol is a major inhibitor of inflammation, but also because inflammatory cytokines can inversely activate the HPA axis and reduce levels of anti-inflammatory cytokines (e.g., IL-4) downstream of the HPA axis, suggesting that microbiota regulation produces the immune hyperactivation and inflammatory responses present in PTSD (Doney et al., 2022).

### 3.3 Immune system

Dysbiosis of the gut microbiota leads to increased gut permeability (Myint et al., 2009) allowing bacterial metabolites and antigens to enter the circulation (Förstermann and Sessa, 2012). These substances trigger immune activation *via* pattern recognition receptors, such as toll-like receptors on the surface of immune cells (Torreilles et al., 1999), inducing the release of pro-inflammatory cytokines such as IL-6, TNF-α and IL-1β. These cytokines contribute to the chronic inflammatory state (Alpert et al., 2021) and exacerbate PTSD symptoms by disrupting the gut-brain axis, as can be observed, for example, by the fact that another downstream acute-phase protein, c-reactive protein, secreted by the inflammatory cytokine IL-6, is significantly elevated in people with PTSD (Miller et al., 2018). Changes in mRNA expression of interleukin IL-1β and IL-6 within the prefrontal cortex have been associated with increased abundance of *Flavobacterium* and decreased abundance of *Pineobacterium*, which also correlates strongly with the severity of social avoidance (Szyszkowicz et al., 2017). Repeated social defeat stress induces an inflammatory bowel environment by altering mucosal barrier integrity and gut microbiota homeostasis (Yadav et al., 2023). It has also been found that gut microbiota dysbiosis directly contributes to increased blood-brain barrier permeability (Braniste et al., 2014; Diaz Heijtz et al., 2011), and gut microbiota imbalances occurring early in life may similarly increase the susceptibility of mother-isolated individuals to PTSD following a traumatic event, which participates in the progression of the disease course by persistently influencing immune system and neurophysiological functioning, making the individual more susceptible to PTSD following a traumatic event, and contributing to the disease (Usui et al., 2021). Gut microbiota have also been shown to affect the response of microglia to signals from the CNS region, thereby affecting the response to pain and inflammation in people with PTSD (Nathalie et al., 2021). Their chronic activation also leads to the release of proinflammatory mediators and reactive oxygen species (Merz et al., 2021), exacerbating neuronal damage and impairing synaptic plasticity, both of which are associated with PTSD symptoms.

A growing body of evidence emphasizes the involvement of the immune system in the pathogenesis of PTSD (Bekhbat et al., 2017; Elder et al., 2019; Vasileva et al., 2019). Patients with PTSD have significantly elevated levels of proinflammatory cytokines (Doney et al., 2022) which, in turn, disrupt neural circuits responsible for emotion regulation, memory consolidation (Yirmiya and Goshen, 2011), and fear-abatement learning (Doney et al., 2022) through systemic and neuroinflammatory responses. At the same time, proinflammatory cytokines affect brain function through multiple pathways. They can activate microglia in the CNS by crossing the blood-brain barrier or by signaling through the vagus nerve (Cai et al., 2023; Jones et al., 2018). This activation rapidly triggers an inflammatory microenvironment that destroys brain regions (e.g., amygdala, hippocampus, and prefrontal cortex) that are closely associated with PTSD. Thus, correspondingly, the immune system shapes the composition and activity of the microbiota (Belkaid and Hand, 2014).

Cytokines can also alter neurotransmitter systems associated with PTSD: proinflammatory cytokines drive the kynurenine pathway (Stefano et al., 2018), which depletes tryptophan required for serotonin synthesis, leading to mood disorders and anxiety (M.-T. Liu et al., 2009), as well as decreasing dopamine production by depleting tetrahydrobiopterin (BH4), a key cofactor in dopamine synthesis (McVey Neufeld et al., 2015). In addition, elevated levels of quinolinic acid, a neurotoxic metabolite of the kynurenine pathway, potentiate the excitotoxicity of glutamate (De Vadder et al., 2018), which may exacerbate cognitive deficits and mood dysregulation in people with PTSD.

### 3.4 Neurotransmitter

Bacteria within the GM have been shown to produce different neurotransmitters. Some of the major neurotransmitters include GABA (*Lactobacillus* and *Bifidobacterium*), norepinephrine (*Escherichia*, *Bacillus*, and *Saccharomyces*), dopamine (*Bacillus*), acetylcholine (*Lactobacillus*), and 5-hydroxytryptophan (*Escherichia*, *Enterococcus*, *Candida*, and *Streptococcus*) (De Vadder et al., 2018; Hendrickson and Raskind, 2016; Lyte, 2013). The metabolic process products of the microbiota also contain a variety of neurotransmitters, including 5-HT and GABA. 5-HT is a classic stress-related neurotransmitter, 80% of which is synthesized by gut microbiota (e.g., *Escherichia*, *Halobacterium*, *Pseudomonas*, *Streptococcus*, *Bifidobacterium*, *Lactococcus*, *Morgellus*, *Klebsiella*, *Propionibacterium*, *Fusobacterium*, *Rossella* and *Prevotella*) Synthesis (Stefano et al., 2018).

Neurotransmitter imbalance is a central mechanism in the pathophysiology of PTSD (Bonaz et al., 2017). Hanscom et al. proposed that reduced 5-HT activity (particularly *via* 5-HT1A receptor dysregulation) in people with PTSD following traumatic brain injury is strongly associated with impaired mood disorders, anxiety, and fear extinction (Hanscom et al., 2021). McVey et al. argued that neuronal dysfunction in GF mice can be colonization reversal, further supporting a critical role for gut-derived 5-HT in neural development and function (McVey Neufeld et al., 2015). Three studies by De Vadder et al. confirmed that the microbiota regulates 5-HT synthesis (De Vadder et al., 2018), suggesting an important role for 5-HT in the pathogenesis of PTSD (Bonnin and Levitt, 2011; Chan et al., 2024; Myint et al., 2009). Interestingly, neurons in GF mice are unable to synthesize 5-HT, and supplementation with a 5-HT4 receptor agonist significantly improves their enteric neurodevelopment (PTSD manifests itself in mice as a state of generalized fear stemming from a glutamate-to-GABA transmitter switch in dorsal lateral flanking neurons of the middle suture bundle, and a similar shift has been observed in *postmortem* brain tissue of people with PTSD (Li et al., 2024). In a rat model of PTSD/alcohol use disorder co-morbidity, Borgonetti et al. concluded that there are sex differences in IL-18 regulation of GABA synapses (Vaiva et al., 2004), and thus inhibition of amygdala GABA neurons is critical for precise regulation of the consolidation, expression, and extinction of fear memories. GABA is an inhibitory neurotransmitter, and classical studies have shown that microbiota downregulation of low plasma GABA levels as a predictor of acute PTSD (Vaiva et al., 2004). The GABA system is involved in the pathophysiological processes of PTSD when functioning is significantly reduced in patients (Huang et al., 2023), and Meyerhoff et al. demonstrated that changes in GABA concentrations in patients with PTSD were consistent with the results of panic disorder and social anxiety disorder (Meyerhoff et al., 2014).

Reduced 5-HT transmission is influenced not only by emotional stability, a characteristic manifestation of PTSD (Liu W.-Z. et al., 2018), but also by the release of short-chain fatty acids (SCFAs) from gut chromaffin cells. SCFAs, stimulate 5-HT receptors located on sensory fibers of the vagus nerve (Fukumoto et al., 2003).

### 3.5 SCFAs

Short-chain fatty acids including butyrate, acetate, lactate and propionate, which are mainly produced by *Bifidobacterium*, *Lactobacillus*, *Rhizobium bradyrhizogenes*, *Kolbachia*, *Roseobacter*, and *E. faecalis* in the colon, have significant neurological effects by modulating neurotransmitter synthesis, inhibiting neuroinflammation, and enhancing the integrity of the blood-brain barrier through the gut-brain axis. Among the aforementioned Gram-positive anaerobes, butyrate-producing bacteria, namely, *E*. *faecalis* in the *Clostridium globosum* group and *Enterobacter cloacae*/*Rose bacillus* in the *C*. *globosum* group, are widely distributed (Mokhtari et al., 2017).

Butyrate is a double-edged sword (Liu H. et al., 2018), and its sodium salt is involved in psychiatric mechanisms through the production of neurotrophic factors (Varela et al., 2015) and the conversion of subthreshold learning events into long-term memories through brain-derived neurotrophic factor-dependent mechanisms (Intlekofer et al., 2013). These findings offer hope for inhibiting the recurrence of traumatic memories in PTSD. An abnormal gut environment (e.g., gut barrier dysfunction, SCFAs concentrations, and a variety of microbial metabolites) also characterizes the pathology of PTSD (Mellon et al., 2019) Veterans with PTSD have a proinflammatory gut environment that includes higher levels of metabolites of microbial origin, such as acetic, lactic, and succinic acids, and gut barrier dysfunction [lipopolysaccharides (LPS) and lipopolysaccharide-binding proteins,] increased HMGB1, along with an increased number of extracellular vesicles of gut epithelial cell origin (Voigt et al., 2022).

### 3.6 Other mechanisms

Initially, it was thought that the gut microbiota might be a viable target for the direct treatment of disorders associated with amygdala dysregulation, including visceral pain, post-traumatic stress disorder, and depression (Cowan et al., 2018; Hoban et al., 2017; Seo and Anderson, 2019; Stilling et al., 2015). Hoban et al. demonstrated for the first time, by means of a genome-wide transcriptome analysis approach, that the presence of the host microbiota is essential for amygdala-dependent memory retention during appropriate Behavioral responses are critical because microbiota-driven modulation of neuronal function can directly lead to fear suppression and dysbiosis learning. It also induces neuronal changes in the amygdala region of the brain, thereby alleviating PTSD symptoms (Hoban et al., 2018). Transmembrane two-photon imaging performed by Chu et al. showed that ablative learning deficits following gut microbiota manipulation in adult mice were associated with deficits in synaptic dendritic spine remodeling and reduced activity of cue-encoding neurons in the medial prefrontal cortex (Chu et al., 2019). Although these important observations highlight the relevance of the gut-brain axis and fear circuits, the mechanisms by which the gut-brain axis directly influences fear and stress circuits remain largely unknown.

PTSD is more common in women than in men (Tolin and Foa, 2006), and previous studies in human and animal models suggest that differences between the ways in which the gonadal hormones testosterone or estrogen interact with the HPA axis or regulate hippocampal function may have contributed to this (Briscione et al., 2017; Fenchel et al., 2015; Lilly et al., 2009; Maeng and Milad, 2015; Richter-Levin et al., 2019) report. Gender is also one of the important host factors influencing the human microbiota (Dominianni et al., 2015; Kim et al., 2020) and the animal microbiota (Elderman et al., 2018; Org et al., 2016). Gender-related differences, such as sex hormones, may interact with gut microbiota, potentially influencing the gut-brain axis and contributing to the development of PTSD.

Interestingly, medical dogs of veterans with PTSD may also influence human health by altering the microbiota of the population, thus potentially providing additional mechanisms to influence human health (Hoisington et al., 2018).

## 4 Intervention strategies for PTSD based on the gut-brain axis

### 4.1 Probiotics

Sudo et al. have shown that in GF mice, exposure to stress induces hyperactivation of the HPA axis, which can be completely reversed by reconstitution with Bifidobacterium infantis. These data further open the possibility that supplementation with diet and/or beneficial bacteria may ultimately influence disordered behavior (Sudo et al., 2004).

Targeting gut microbiota, a potential key modulator of the immune and nervous systems, could lead to greater improvement in mood symptoms in patients with depression or anxiety. The composition and function of the gut bacterial community can be improved through dietary interventions or the use of beneficial bacteria such as probiotics. Although, in this regard, clinical trials have not been adequate in terms of design or number of subjects involved. However, studies that have been conducted have shown that administration of different species of *Lactobacillus* and *Bifidobacterium* is associated with improved mood and reduced anxiety (Benton et al., 2007; Rao et al., 2009). This is especially true in subjects with low cortisol levels (Messaoudi et al., 2011). In addition, taking fermented dairy products containing probiotics affects activity in brain regions that control central processing of emotions in women (Tillisch et al., 2013). In addition, two intervention studies for PTSD examined the reported effects of microbiota-targeted supplements on PTSD symptoms in veterans (Brenner et al., 2020; Gocan et al., 2012). The first study (Gocan et al., 2012), of 10 veterans with PTSD, found that regular consumption of a fermented soy preparation (FSWW 08) for 6 months reduced anxiety and panic. However, causal inferences from this study were limited by the lack of a control group as well as small sample size, selection factors, and reporting bias. In the second study (Brenner et al., 2020), participants were randomly assigned to either the intervention group or the placebo group (once daily for 8 weeks ± 2 weeks) in a 1:1 ratio, stratified according to irritable bowel syndrome status. Plasma C-reactive protein (CRP) concentrations tended to decrease in the probiotic-supplemented group compared with the placebo group, and the stress response was more pronounced in the placebo group. Brenner et al. also conducted a systematic review to evaluate existing studies on prebiotic and probiotic interventions in patients with traumatic brain injury and PTSD (Brenner et al., 2017).

### 4.2 Dietary interventions

Because dietary interventions can be effective in ameliorating the effects of chronic inflammation, medical associations have identified them as first-line and/or adjunctive tools for many neurodegenerative diseases (Parkinson et al., 2023), which is also an important avenue for psychiatric disorders, particularly PTSD (Lee et al., 2022). In addition, dietary interventions may promote positive neuroplasticity (Sugden et al., 2024), strengthen the gut-brain axis system (Campaniello et al., 2022), reduce the effects of neuroinflammation, and increase the window of tolerance. Leclercq et al. have proposed that it is possible to target abnormalities in these systems through manipulation of certain gut bacterial communities, either directly through supplementation or indirectly through dietary and other novel approaches (Leclercq et al., 2016). Two recent feasibility studies have shown that it improves PTSD symptoms. Herbert et al. selected 10 U.S. veterans with PTSD and chronic pain and gave them a plant-based diet high in dietary fiber for 2 weeks, followed by 2 weeks of a regular diet. The veterans reported improvements in both chronic pain and PTSD symptoms (Herbert et al., 2023). Arcan et al. studied responders to the World Trade Center disaster. Responders either received nutritional counseling or help from the Mediterranean diet. People on the Mediterranean diet showed greater changes in the Posttraumatic Checklist-dsm -5 (PCL-5) (Arcan et al., 2024).

People with PTSD also need to be aware of the intake of fast food as well as other ultra-processed foods, as ultra-processed foods increase inflammatory processes in the gut (Tristan Asensi et al., 2023) and may also promote neuroinflammation (Firth et al., 2019), and a key component of ultra-processed foods is the lack of dietary fiber. Conversely, diets rich in dietary fiber promote certain types of healthy bacteria (i.e., *Bifidobacterium*, *Lactobacillus*, *Tricholobacteriaceae*, *Cyanobacteria*, *Coccidioides faecalis*, *Roseobacteria* and *E. faecalis*) that are able to break down complex carbohydrates into short-chain fatty acids through fermentation (So et al., 2018), potentially helping to alleviate PTSD.

### 4.3 Natural medicines targeting the gut microbiota for PTSD

In the treatment of PTSD, the only drugs that target the treatment of people with PTSD and animal models *via* the gut-brain axis are the natural medicines cannabis, yellow essence, and jiawei xiaoyaoshan, with more discussion focusing on the broad neuroprotective effects of natural medicines (including marine natural products and plant natural products) (Fakhri et al., 2021; Shimizu et al., 2015), prevention of apoptosis, and the effects of oxidative stress (Baek and Kim, 2020; Xing et al., 2020).

Cannabinoids alter the gut microbiota of people with PTSD by modulating fear memory and influence n-3 polyunsaturated fatty acid metabolic pathways (Okubo et al., 2018). A few years ago, McLaughlin et al. suggested that endogenous cannabinoid signaling systems in the medial frontal cortex, a key brain region for fear memory, may be attractive targets for the treatment of stress-related disorders (McLaughlin et al., 2014). In recent years, it has been found that such drugs also modulate GABA receptor activity and cortisol levels *via* the gut-brain axis (Fraguas-Sánchez and Torres-Suárez, 2018) to enhance psychological resilience (B. Gao et al., 2023). Research suggests that cannabinoids may play a potential therapeutic role in PTSD-related symptoms by modulating gut microbiota and the gut-brain axis. In contrast, treatment of Chronic Unpredictable Mild Stress (CUMS)-induced mice with flavonoid polysaccharides increased the relative abundance of Muribaculaceae, Dubosiella, and *Lactobacillus*, and decreased the relative abundance of *Akkermansia*, *Helicobacter*, and *Clostridium methylpentosum* relative abundance (Shen et al., 2022), which may regulate oxidative stress and nlrp3-mediated inflammation in a Nrf2/HO-1 signaling pathway-dependent manner, thereby preventing sps-induced ptsd-like behavior and synaptic damage (Xie et al., 2024). In addition, the herbal medicine jiaweixiaoyaosan, which acts as a complex of natural medicines that can influence the composition of the gut microbiota, has also been reported to alleviate ptsd-related symptomatic aspects by regulating the improvement of the HPA axis and hormonal disorders, increasing neurotransmitter content, neurogenesis, and modulating the synthesis of related enzymes (Xie et al., 2023).

## 5 Perspective

This review explores the potential role of gut microbiota in the development of post-traumatic stress disorder (PTSD) from several perspectives, and remains wary of the limitations of extrapolating its conclusions to human studies in terms of animal modeling. On the one hand, there are essential differences in the composition of gut microbiota between species, resulting in the abundance and function of certain bacteria not being consistent between humans and animals; on the other hand, acute, single stress stimuli (e.g., restraints, electric shocks, *etc.*) are often used to simulate PTSD in animal models, whereas actual clinical traumatic events are often complex, diverse, and involve long-term psychological and socio-environmental factors, making it difficult to replicate them exactly. In addition, most animal experiments use homozygous strain individuals, ignoring genetic background and lifestyle differences in human populations. Therefore, animal studies on PTSD and gut microbiota should remain cautious in the interpretation of results and extrapolation of mechanisms. With regard to population studies, little is still known about the longitudinal dynamics of gut microbiota in people with PTSD. Most of the studies used cross-sectional design, which makes it difficult to reveal the temporal evolution of the microbiota at different stages before, during, and after trauma and its potential causal links with changes in clinical symptoms. In the future, there is an urgent need to conduct longitudinal follow-up studies based on large samples, combined with gender stratification and multi-omics technology, to comprehensively depict the key nodes and mechanisms of gut microbiota in the occurrence, development and recovery of PTSD, so as to provide theoretical support for individualized treatment.

Currently, the neurobiological mechanisms of PTSD are regulated by gut microbiota, and future research could be further expanded in the following directions: to develop therapeutic strategies that precisely target the gut-brain axis. Currently, the main pharmacological treatment for PTSD is based on selective 5-hydroxytryptamine reuptake inhibitors (e.g., sertraline, paroxetine), and there is still a lack of research exploring whether gut microbiota can enhance its efficacy or serve as an adjunctive therapeutic target. Promoting clinical research on probiotics and fecal transplants: Preliminary studies have suggested that probiotics and Fecal Microbial Transplantation (FMT) may have therapeutic potential in mood disorders, but their long-term efficacy, strain-specific selection, timing of interventions, and individualized response mechanisms in PTSD need to be systematically validated in large clinical cohorts. Standardization of probiotic clinical application and scientific education: Currently, there is excessive expectation or misuse of probiotic efficacy in the general public and in some clinical practices, with vague intervention goals, random strain selection, and a lack of unified regulatory standards, which limits its scientific promotion. In the future, we should strengthen the research on the joint intervention mechanism of diet and probiotics, clarify its indications and mechanism, promote the formulation of regulatory standards at the policy level, and strengthen public health education.

In addition, attention needs to be paid to the potential of traditional medicine and natural medicines: natural medicines may affect the gut-brain axis by modulating the inflammatory response, maintaining neurotransmitter homeostasis, and improving the gut barrier function. Although it has been reported in the literature that drugs such as Cannabis sativa, Rhizoma Polygoni Multiflori and Jiawei Yiwu San have some interventional effects on PTSD, there is a lack of systematic research to elucidate their mechanisms of action and targets of intervention. In the future, we should explore the precise pathways of natural drugs to regulate the gut microbiota and central nervous system function.

This review still has some limitations. There is a large heterogeneity in the literature included in this paper in terms of study population, gut microbiota testing methods, and PTSD modeling approaches (e.g., population studies vs animal models), which limits cross-sectional comparisons and comprehensive interpretation of results. Most studies were cross-sectional in design, making it difficult to clarify the causal relationship between gut microbiota and PTSD. In addition, the lack of uniform quantitative indicators and intervention evaluation systems also prevented meta-analysis in this review, affecting the systematic summary of the strength of evidence.

## 6 Conclusion

In recent years, with the deepening of gut-brain axis research, more and more evidence suggests that gut microecology plays an important role in the occurrence and development of post-traumatic stress disorder (PTSD). In this review, we systematically reviewed the current research progress on the association between gut microbiota and PTSD, and summarized the potential mechanisms by which the gut-brain axis may affect PTSD in terms of inflammatory response, neurotransmitter metabolism, HPA axis regulation and immune pathways. Meanwhile, we summarize the current major microecological intervention strategies, including probiotics, prebiotics, dietary modification, and FMT, and discuss their potential value in alleviating PTSD symptoms.
